# Supplementary material for: Checkpoint Inhibitor-Associated Scleroderma and Scleroderma Mimics
Source: Pharmaceuticals (Basel). 2023 Feb 8;16(2):259. doi: 10.3390/ph16020259 (PMC9962184; doi:10.3390/ph16020259)
Supplement: Supplementary file 1 [file pharmaceuticals-16-00259-s001.zip › pharmaceuticals-2170743-supplementary.pdf]

Supplemental Table S1: Summary statistics calculated from cases in review			
	**Cases of ICI-SSc (n= 4)**	**Cases of ICI-Morphea (n= 5)**	**Cases of ICI-Eosinophilic fasciitis (n= 14)**
**Median age***	66 years	53 years	56 years
**Malignancy types**	Melanoma 75%<br>Lung 25%	Melanoma 80%<br>Colorectal cancer 20%	Melanoma 64%<br>Lung 21%<br>Breast 7%<br>Bladder 7%
**ICI use**	0%	60%	14%
Combination ICI	75%	20%	71%
Anti-PD-1	25%	0%	14%
Anti-PDL1	0%	20%	0%
Anti-PDL1 and Other			
**Median onset after ICI start****	47 weeks	30 weeks	56 weeks
**Median duration of ICI-SSc****	32 weeks	30 weeks	26 weeks
**Treatments used**	Steroids: 75%<br>Hydroxychloroquine: 25%<br>IVIG: 25%<br>Mycophenolate: 50%	Topical steroids: 60%<br>Systemic steroids: 60%<br>Colchicine: 20%<br>Cyclophosphamide: 20%<br>Rituximab: 20%<br>Hydroxychloroquine: 20%<br>Calcipotriol: 20%	Systemic steroids: 93%<br>Methotrexate: 57%<br>Cyclosporine: 7%<br>IVIG: 14%<br>Abatacept: 7%<br>Infliximab: 7%<br>Omalizuman:7%<br>Mycophenolate: 14%

*Excluding non-exact ages, **When known
CTLA4: cytotoxic T-lymphocyte-associated protein 4ICI: Immune checkpoint inhibitor, ICI-SSc: Immune checkpoint inhibitor associated systemic sclerosis, PD1: Programmed cell death protein 1, PD-L1: Programmed death-ligand 1
